# Supporting the Sharing of Mental Health Challenges in the Workplace: Findings from Comparative Case Study Research at Two Mental Health Services

**DOI:** 10.3390/ijerph182312831

**Published:** 2021-12-06

**Authors:** Alicia Jean King, Tracy Lee Fortune, Louise Byrne, Lisa Mary Brophy

**Affiliations:** 1 Department of Occupational Therapy, Social Work and Social Policy, La Trobe University, Melbourne 3086, Australia; t.fortune@latrobe.edu.au (T.L.F.); l.brophy@latrobe.edu.au (L.M.B.); 2 School of Management, College of Business and Law, RMIT University, Melbourne 3001, Australia; louise.byrne3@rmit.edu.au; 3 Program for Recovery and Community Health, Department of Psychiatry, Yale School of Medicine, New Haven, CT 06511, USA; 4 Melbourne School of Population and Global Health, The University of Melbourne, Melbourne 3010, Australia

**Keywords:** MH stigma, occupational health psychology, leadership, work teams, organizational culture, MH services, employee attitudes, job performance, inclusion, diversity

## Abstract

Personal experience with mental health (MH) challenges has been characterized as a concealable stigma. Identity management literature suggests actively concealing a stigma may negatively impact wellbeing. Reviews of workplace identity management literature have linked safety in revealing a stigma to individual performance, well-being, engagement and teamwork. However, no research to date has articulated the factors that make sharing MH challenges possible. This study employed a comparative case study design to explore the sharing of MH challenges in two Australian MH services. We conducted qualitative analyses of interviews with staff in direct service delivery and supervisory roles, to determine factors supporting safety to share. Workplace factors supporting safety to share MH challenges included: planned and unplanned “check-ins;” mutual sharing and support from colleagues and supervisors; opportunities for individual and team reflection; responses to and management of personal leave and requests for accommodation; and messaging and action from senior organizational leaders supporting the value of workforce diversity. Research involving staff with experience of MH challenges provides valuable insights into how we can better support MH staff across the workforce.

## 1. Introduction

Personal experience with mental health (MH) challenges has been characterized as a concealable stigma, as it is not always evident to those within one’s social circle [1]. Recent research suggests actively concealing a stigma may negatively impact wellbeing [1,2].

Workplace identity management research has linked safety in revealing other concealable stigmas (e.g., sexual orientation, HIV status, having been the victim of abuse) to performance, well-being, engagement and teamwork [3]. Jones and King’s [3] model contends that organizational, supervisory and individual factors contribute to decisions to reveal a stigma and the outcomes of doing so. The outcomes of revealing MH challenges, specifically, in the workplace, are mixed [4,5,6]. A recent review suggested fear of stigma and discrimination as a major barrier to “disclosure” of MH concerns in any workplace [7].

Edmonson [8] asserts “cultures of silence” in workplaces have implications for teamwork, customer experience and outcomes. Similarly, the “culture of non-disclosure” in MH workplaces around professionals’ own MH challenges [9], is seen to negatively impact service user experiences [10,11,12].

The literature to date suggests factors supporting MH professionals to be more open about their mental health challenges in the workplace, are complex and poorly articulated [13]. A scoping review conducted by the authors found only 23 studies considering sharing with colleagues and supervisors [13]. Existing research particularly focused on reluctance to “disclose” as a barrier to help-seeking, rather than its potential to create more inclusive and safe mental health services [13]. A recent review of “disclosure” by health care professionals (HCPs) identified the need for research “to determine organizational strategies to improve disclosure, as well as to foster more supportive climates for HCPs with mental health difficulties” [14] (p. 11).

This study addressed the question: What makes it possible for MH professionals to share their lived experience (LE) in the MH workplace? Similar precursors and consequences of revealing experience with MH challenges were found and have been identified in relation to other stigmas [3]. Additionally, the role of designated lived experience positions, professional habitus [15] and team culture were evident. Modifiable organizational, supervisor and team factors may lead to improved staff wellbeing, satisfaction and performance.

Please note, we have used the term “revealing” when referring to disclosure, and “sharing” to denote the sharing of experiences with MH challenges and knowledge gained, as recommended by professionals with LE [16].

## 2. Materials and Methods

In order to identify workplace factors impacting sharing, we employed a multiple case study design [17,18] to compare the perspectives and experiences of staff from two MH services in Victoria, Australia [19]. Organization A is a publicly funded, statutory service, providing multidisciplinary MH interventions and support coordination, in clinic, inpatient and home settings. Organization B is a non-government, community MH support service, providing non-clinical psychosocial support and support coordination in home and community settings. Both services work with people experiencing significant and prolonged MH challenges and associated disability.

Case study research has been described as conducting “an in-depth exploration from multiple perspectives of the complexity and uniqueness of a particular […] policy in a ‘real life’ context” [18]. To capture a range of perspectives, staff were purposively sampled for lived experience and work role from the following groups:

- MH professionals identifying with LE, publicly or privately (MHPLE);
- MH professionals not identifying with LE (MHP);
- staff in designated LE roles (Peer Workers);
- staff in supervisory roles (Supervisors) from the above groups.

Description of organizational features was further supported by document analysis [19] of internal and publicly available policies, procedures and position descriptions, relevant to the topic.

Two service-wide e-mails were sent by a senior manager at each organization and people interested in participation contacted A.K. directly. During screening, participants were asked to self-identify if they had personal LE. Participants who had experience supporting or caring for someone experiencing MH challenges (“supporter” LE) only were not included in the MHPLE group.

Given the exploratory nature of the study, qualitative data collection and analysis methods were employed. Individual interviews, using a semi-structured interview guide [20], were audio-recorded, then transcribed, and manually coded by A.K. using NVivo 12 Software [21,22]. A summary of each interview was sent to the relevant participant for member checking. Second stage coding resulted in pattern codes [22] which were compared, across informant groups, using matrix coding queries [21] and displays [17,19]. As described by case study experts [18], an abductive approach was adopted in the final stage of analysis, “a creative inferential process aimed at producing new hypotheses and theories based on surprising research evidence” [23] (p. 167).

The research was guided by an advisory group that comprised 50% LE membership, including representatives from participating organizations, MHPLE, MHP and peer workers. Field notes and journaling were used by A.K. to support reflexivity and bracketing in relation to her experiences as a MHP [24].

The study was approved by the Ethics Committee of La Trobe University (protocol code 2020.041) and by the institutional review boards of participating organizations.

## 3. Results

### 3.1. Participant Characteristics

Thirty-three participants, including 26 women, five men and two non-binary persons, are shown by informant group and organization in Table 1.

Participants had worked in MH services on average 8.23 years (range = 0.5 to 25 years) and for their current employer on average 5.09 years (range = 0.5 to 15 years).

Table 2 shows the types of training in MH related disciplines participants had completed, started and did not complete, or were currently undertaking. Participants from the MHPLE group at Organization A tended to have completed bachelor level degrees (e.g., social work, occupational therapy), qualifying master’s level degrees and other post-graduate (e.g., clinical psychology, psychiatric nursing, psychiatry) tertiary education. Participants from the MHPLE group at Organization B were more likely to have completed a bachelor’s degree, mental health related vocational training (e.g., diploma of community services) or be working towards a tertiary qualification. The MHPLE group at both sites included a small number of staff without training in MH related disciplines employed in service-user facing or operational roles influencing service delivery.

The following findings describe the perspectives and experiences of participants across informant groups, organizations, and professional backgrounds. All names are pseudonyms.

### 3.2. Contextual Factors Supporting Revelation of Mental Health (MH) Challenges

Factors supporting staff to reveal their LE, privately or publicly, in the workplace included organizational, supervisory, and individual factors.

#### 3.2.1. Perceived Organizational Support

Organizational support for LE was judged by participants before and during their employment. MHPLEs at both organizations reported revealing LE in their job interview, describing it as a “qualification” or relevant experience supporting their application: “I don’t think I did that well in the interview, but I think that [revealing LE] might have got me over the line. […] [Organization B] kind of walk the talk on it, a bit” (Tanveer, MHPLE, Organization B). Conversely, concealment of LE was associated with fears about impacts on future career progression.

“I was looking at peer work roles and, in the end, decided against it because I wasn’t really comfortable with the level of disclosure that is implicit in the peer role. I was really reluctant to have that on my resume.”

(Finley, MHPLE, Organization B)

#### 3.2.2. Perceived Supervisor Support

Supervisors at both organizations supported staff “privately” during periods of MH challenges. However, support for sharing LE “publicly” in the workplace was less consistent. Some participants had opportunities to reflect upon their sharing in supervision.

“My supervisor […] creates a safe space for me to be able to explore those issues […] so that, […] I’m able to […] develop my relationship with LE and sharing in a way that supports the work and our consumers, as well.”

(Yarden, MHPLE, Organization B)

Others felt discouraged from discussing their LE: “So, I’d actually like to sometimes be able to share some of those things. And I feel like it’s a bit, ‘Oh, let’s not go there.” (Melissa, MHPLE, Organization A)

#### 3.2.3. Individual Differences

The key factor differentiating individuals who shared “publicly’’ seemed to be their habitus [25] or way of being in the workplace, specifically, their preference for “being open” above perceived “professionalism:” “I’m pretty real and raw, at times. […] I do know that I can be a bit too honest and open, for some people” (Pragun, MHPLE, Organization A). Participants described being “authentic” as valuable in “connecting” with others and improving services.

“By being myself fully, I felt it might allow space for other people to feel they can be themselves fully, […] feeling permission to be themselves and to be their authentic selves…. can create… contribute to the change that is maybe needed.”

(Tanveer, MHPLE, Organization B)

Although there were participants at Organization A who felt similarly, they were conscious of violating professional imperatives around “self-disclosure:” “It’s trying to be neutral… trying to blank slate. Which I don’t agree with because I don’t think we can separate our LE and our politics and our ethic…” (Melissa, MHPLE, Organization A)

Senior professionals’ sense of having “less to lose’’ was tempered by the responsibility of modelling “safe” identity management.

“The older you get, and the more senior you get in your position, the more people value what you say […]. When you […] do disclose something, that it carries a lot of weight. And so, it’s important to be very careful about how you do that and judicious.”

(Quinn, MHPLE, Organization A)

### 3.3. Identity Management Behaviours in the Workplace

Members of all informant groups reported concealing MH challenges in the workplace, with the goal of avoiding prejudice and discrimination. Descriptions of concealment by MHPLE reflected the additional cognitive load this created: “The art form for me is how do I do that without letting them know that I’m speaking from my own LE? Because I effectively remain hidden and choose to… believe I need to.” (Kevin, MHPLE, Organization A).

In circumstances where MHPLE were unsure if their revelation would be received positively or negatively, there was a tendency to engage in “signaling” [3] behaviors, aimed at assessing the safety of revealing: “So, I tried to say that some of the clinicians […] might have their own issues but not specifically say it was my own issues.” (Amanda, MHPLE, Organization A).

The majority of MHPLE reported “revealing” [3] their LE within the private conversations with supervisors or “close” colleagues. Peer workers, and a small number of MHPLE, used the “umbrella of LE” when sharing “publicly.” More stigmatized experiences (e.g., substance abuse) or diagnoses (e.g., personality and psychotic disorders) were revealed within the safety of relationships with trusted confidantes, sometimes with the goal of challenging stigma.

“I think, it is good to share so they can see someone that’s going to work and seeming, relatively, fine… and, having that diagnosis. It’s part of breaking down the stigma.”

(Neeru, Peer worker, Organization A)

### 3.4. Sharing Lived Experience

Beyond identity management behaviors (e.g., concealing, signaling, revealing), participants also shared knowledge they had gained through LE. For MHPLE, revealing experience with MH challenges was a precursor to sharing, described as “starting” or “going deeper” in “a conversation.” MHPLE at Organization A were noted to use the term “normalize” in this context: “To… normalize… to say, ‘You’re not the only one having this problem. I’ve gone through this, too. […] These are the symptoms that I had […] and the strategies that helped me get through that’” (Kyra, MHPLE, Organization A). For peer workers the sharing of LE was described as an “expectation,” that was not always met positively: “I’m there to say stuff like that but then what happens with what I say? It just gets tossed aside” (Abby, Peer worker, Organization A)

### 3.5. Team Culture and Type of Sharing

The distinguishing factor between sharing LE “publicly” and “privately” appeared to exist at a team level. “Public” sharing occurred in teams described as non-hierarchical and supportive of discussions of “struggles”.

“We are a multi-disciplinary team but there’s no hierarchy at all. It’s a very flat structure. [...] there’s no person or professional on the team is more or less than anyone else. [...] and I’ve only received encouragement to […] discuss issues, and consumers, and struggles with the team.”

(Halcyon, Peer worker, Organization B)

In contrast, staff in other teams described experiences with “bullying” or “defensiveness” that discouraged sharing: “There’s people in the office that don’t like other people and people feeling intimidated by someone else or bullied. […] So, people don’t share. I feel, sometimes, they’re quite defensive.” (Neeru, Peer worker, Organization A).

Participants at both organizations had witnessed a change in their team culture, with a change of manager, particularly when coupled with explicit efforts to change team culture.

“Most people are okay with looking at… at naming their strengths and weaknesses and working together around that… which is quite challenging, really. […] It’s hard but we’re getting really good at it. It’s a great environment now, and necessary for the work. Like, we’ve got a great healthy team… really supportive team.”

(Blake, Peer worker, Organization B)

### 3.6. Consequences of Revealing and Sharing Lived Experience (LE)

Participants identified a range of impacts associated with sharing. For the purpose of this paper, the consequences of sharing within supportive relationships and teams will be discussed in terms of three key themes: “lifting the burden;” “sharing the load;” and “bringing my whole self to work.”

#### 3.6.1. Lifting the Burden

The most prominent impact of sharing was the benefit to participants’ emotional wellbeing. Participants described MH challenges as a “weight,” “burden,” or “load” that was “relieved” or “lifted” when shared: “I, definitely, feel that talking about it is quite… healing and … it definitely, lightens the load” (Pragun, MHPLE, Organization A). This impact was reported in relation to “public” and “private” sharing.

#### 3.6.2. Sharing the Load

Sharing with trusted confidantes resulted in receiving emotional and practical support in the workplace that assisted participants in managing work demands: “I think, like, just being there makes [MHPLE] function a bit more… better… probably they feel they can come and talk to you” (Kyra, MHPLE, Organization A). MHPLE who received support reported it lengthened their tenure: “One of the reasons I’ve stayed here, is because of that culture” (Ryker, MHPLE, Organization A).

Unidirectional support over time, however, had the potential to leave MHPLE feeling like “a burden:’’ “I just feel like I shouldn’t be her issue anymore. Like, I feel like a burden for her, I guess” (Lauren, MHPLE, Organization A). Whereas mutual sharing and support allowed MHPLE to feel “equally” valued: “And, I guess probably, that put us on equal footing. […] We definitely support each other in that way. When things get really difficult, we’re bouncing off of each other” (Sasha, MHPLE, Organization B). Mutual sharing among colleagues also resulted in practical support.

“So, the ones that you care about, and they care about you, you actually would go out of your way to help them […]. If you’re closer to someone they naturally volunteer to help you more and you feel more supported. If you don’t have that, it’s just stressful.”

(Melissa, MHPLE, Organization A)

Benefits to performance occurred with “private” sharing but were amplified where staff felt able to “navigate challenges’’ openly, as a team.

#### 3.6.3. Bringing My Whole Self to Work

Participants who shared openly also reported increased job satisfaction related to a sense of authenticity in the workplace.

“I guess, what’s developed from that is a feeling of real satisfaction within my role because I feel like I can bring all of myself to this work. It’s not like I have to put on a professional façade and feel like I have a whole lot of experience that might be informing the way I do my work but that I need to keep a secret.”

(Yarden, MHPLE, Organization B)

#### 3.6.4. Once Bitten, Twice Shy

Negative consequences reported by participants, were mostly related to preoccupation with the fear they had “overshared,” shared unnecessarily, or that information shared might be “used against them.” Experiences of being “burnt” in past supervisory relationships and workplaces made them cautious about sharing: “I had one manager who, actually, used to use it against me. That manager isn’t working anymore but… um… it did a lot of damage” (Ione, MHPLE, Organization B).

## 4. Discussion

The findings of this research contribute three novel insights into the sharing of LE by MH professionals in the workplaces studied.

Firstly, peer workers’ comments reveal the impact of organizational differences in supporting sharing. At Organization A, a culture of silence around MH challenges restricted peer workers’ sharing, despite it being an expectation of their role. In contrast, Organization B, which made LE-informed training and supervision available to staff outside of designated roles, saw improvements across the workforce in sharing for purposes other than help-seeking. This supports the value of whole-of-workforce approaches [26], in “cultivating spaces for sharing” (Orlando, MHPLE, Organization B). Similarly, “messaging” from senior leadership supporting the value of LE endorsed participants’ own belief in their experiences as an asset to their work.

The second novel contribution of this research relates to the influence of individual dispositions to sharing. New approaches to professional work, in the context of service reform, came into conflict with traditional notions of “professionalism” for MHPLE. Bordieu’s [25] description of a “cleft habitus” or “habitus divided against itself” (p. 511) may be a useful way of framing these findings. MHPLE in this study highlighted a lack of support in navigating these tensions. Nairz-Wirth and Feldmann [15], described teachers who successfully managed these tensions as having a “hybrid habitus” which helped them to adapt within different work contexts.

**Figure 1:**
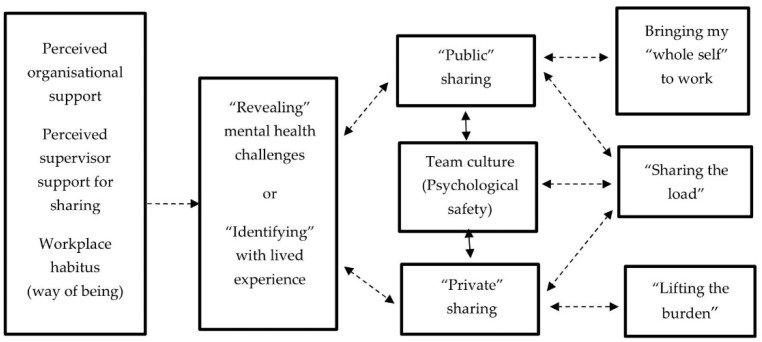
Factors supporting and consequences of sharing in supportive relationships and teams.

Thirdly, these findings show the influence of team culture on how staff shared. All MHPLE and peer workers shared in private conversations with trusted colleagues and supervisors but only some felt safe to share “publicly” in the workplace. Figure 1 shows the relationship between team culture, the type of sharing participants engaged in (e.g., “public” or “private”), and the consequences of sharing.

Team cultures described by participants as supportive of open sharing demonstrated similarities with descriptions of “psychological safety,” that is, “…a climate in which people are comfortable expressing and being themselves” (p. xvi) [8]. Edmonson [8] describes the impact of such a culture as “taking off the brakes that keep people from achieving what is possible” (p. 21).

The tools for cultivating sharing more consistently across teams appear to be within the hands of the organisations studied. Examples of practices supporting sharing included:

- planned and unplanned, individual and team “check-ins”;
- mutual sharing and support by colleagues and supervisors;
- opportunities for individual and team reflection.

Furthermore, frameworks for co-reflection and peer support, used by staff in designated LE roles, offered guidance in breaking down hierarchies that erode psychological safety [8].

Whilst case study research does not aim for generalization, cross case analysis, with reference to existing explanatory models [17], suggests the following implications may be transferable to other contexts.

Specific to the MH workplace, research regarding disclosure of MH challenges by professionals has often focused on its role in help seeking [27,28,29,30] and access to accommodation [31]. Important issues to address [32], the framing of staff MH challenges, exclusively in terms of impairment and equity, represents a barrier to MH service reform. The findings of this research add to the growing recognition that concealment of MH challenges perpetuates stigma beliefs in the MH workforce [9,33]. Unlike other workplaces, the LE of staff with MH challenges constitutes a resource for service innovation and growth, largely untapped to date.

Within and beyond the MH setting, this research supports the application of Jones and King’s [3] model to disclosure of MH challenges. The assertion that active concealment has implications for well-being [1,3] is also supported. Similarities between our findings and the results of quantitative workplace stigma research highlight potential downstream effects of concealment on work performance, satisfaction and teamwork but, also, the vital role of colleagues, supervisors and employers in supporting staff to share.

In considering the transferability and dependability of these findings, it should be noted that challenges in recruiting MHP and achieving gender balance were encountered. Furthermore, the relative absence of negative consequences experienced by participants should be interpreted with caution, given participants’ reported selectivity in sharing. Future research employing psychological safety approaches [8] may provide a way forward beyond existing stigma reduction approaches.

## 5. Conclusions

The sharing of LE in the MH workplace is a complex and largely unexplored phenomenon with implications for individual and team performance. This research demonstrates that efforts to include LE perspectives, without attention to workplace factors supporting sharing, are unlikely to realize their aims. Resonance between our findings and the wider organizational research literature suggest the salience of identity management [3], professional habitus [15] and psychological safety [8] constructs, in tackling the barriers to sharing. Creating workplaces where staff are able to “bring their full selves to work” [8] (p. 11) may hold the key not just to improving staff MH and well-being but improving service user experiences of support.

## Figures and Tables

**Table 1:** No. of participants by informant group and organization (*n* = 33).

	MHPLE	MHP	Peer Workers	Supervisors ^1^
Organization A	10	1	4	8
Organization B	11	4	3	6

^1^ From any other informant group.

**Table 2:** No. of participants by training in MH related disciplines (*n* = 33).

Discipline	No. of Participants
Social Work	10
Non-clinical, MH related ^1^	9
Nursing	5
Psychology	5
Other clinical ^2^	5
Peer Support	5
Occupational Therapy	2
Psychiatry	2

^1^ E.g., community services, counselling. ^2^ E.g., family therapy, narrative therapy.

## Data Availability

The data presented in this study are available on request from the corresponding author. The data are not publicly available due to privacy reasons.
